# Supplementary material for: Multimorbidity and Complex Multimorbidity in India: Findings from the 2017–2018 Longitudinal Ageing Study in India (LASI)
Source: Int J Environ Res Public Health. 2022 Jul 26;19(15):9091. doi: 10.3390/ijerph19159091 (PMC9332385; doi:10.3390/ijerph19159091)
Supplement: Supplementary file 1 [file ijerph-19-09091-s001.zip › ijerph-1764743-supplementary.pdf]

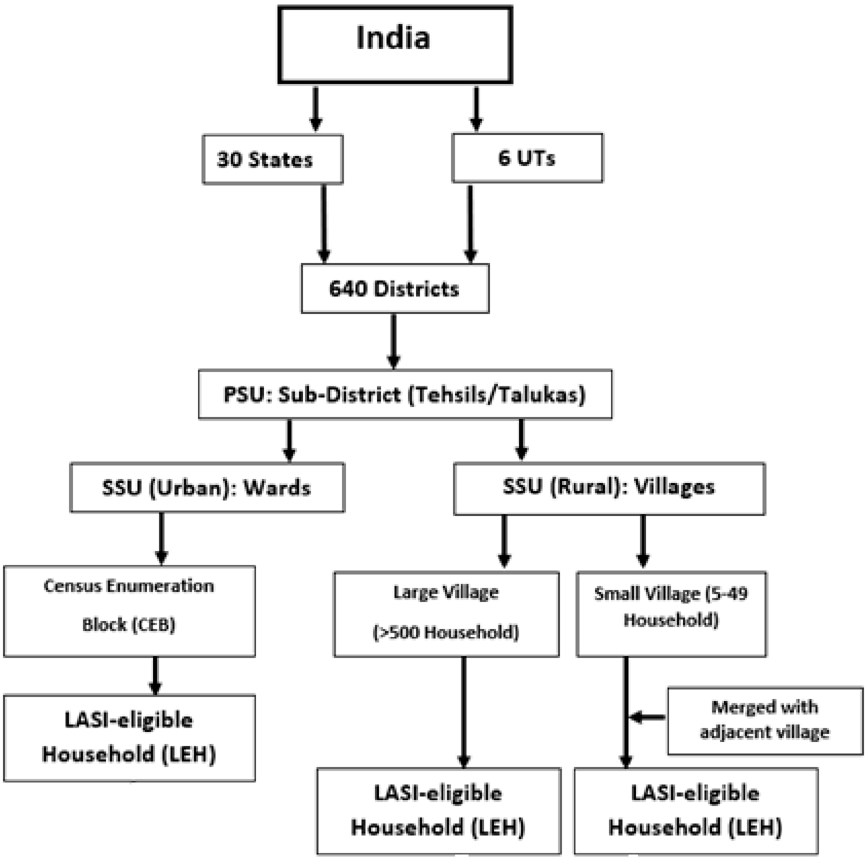

Figure S1. Description of sampling process adopted by Longitudinal Ageing Study in India (LASI).

Table S1. Prevalence of chronic conditions among study population.

List of Chronic Conditions	Frequency (n)	Percentage (%)
Hypertension	14621	47.96
Diabetes	6746	22.13
Cancer	340	1.12
Chronic lung disease	3507	11.50
Chronic heart disease	2151	7.06
Stroke	1027	3.37
Chronic bone/joint conditions	8883	29.14
Psychological or neurological conditions	1281	4.20

Hypercholesterolemia	1373	4.50
Thyroid disorders	1581	5.19
Gastrointestinal problems	9245	30.32
Skin disease	2710	8.89
Chronic kidney disease	379	1.24
Urine incontinence	1679	5.51
Oral conditions	18465	60.56
Vision problems	2379	7.80
Hearing Problems	3224	10.58
Obesity	13136	43.64

Table S2. Commonly occurring patterns of chronic conditions among the non-complex multimorbidity group (Prevalence>1%).

Pattern	Frequency	Percentage
	n	%
Obesity, Oral conditions	1581	7.92
Gastrointestinal problems, Oral conditions	1418	7.1
Chronic bone/joint conditions, Oral conditions	1071	5.36
Hypertension, Oral conditions	1060	5.31
Obesity, Hypertension	1046	5.24
Obesity, Hypertension, Oral conditions	522	2.61
Obesity, Hypertension, Diabetes	511	2.56
Hypertension, Diabetes	434	2.18
Obesity, Gastrointestinal problems	418	2.09
Hypertension, Gastrointestinal problems	3867	1.94
Obesity, Chronic bone/joint conditions	379	1.9
Hypertension, Gastrointestinal problems, Oral conditions	371	1.86
Chronic lung disease, Oral conditions	347	1.74
Oral conditions, Hearing Problems	341	1.71

Obesity, Diabetes	335	1.68
Obesity, Chronic bone/joint conditions, Oral conditions	327	1.64
Skin disease, Urine incontinence	324	1.62
Obesity, Gastrointestinal problems, Oral conditions	301	1.52
Hypertension, Chronic bone/joint conditions	291	1.46
Diabetes, Oral conditions	289	1.45
Chronic bone/joint conditions, Gastrointestinal problems, Oral conditions	270	1.35
Oral conditions, Vision problems	238	1.19
Chronic bone/joint conditions, Gastrointestinal problems	206	1.03
Obesity, Hypertension, Chronic bone/joint conditions	203	1.02

Table S3. Commonly occurring patterns of chronic conditions grouped by ICD-10 chapters among complex multimorbidity group (Prevalence >1%).

ICD 10 Chapters	Pattern	Frequency	Percentage
		n	%
IV, IX, XI	Endocrine / Nutritional / Metabolic system, Circulatory system, & Digestive system	1398	13.28
IX, XI, XIII	Circulatory system, Digestive system & Musculoskeletal/connective tissue	975	9.26
IV, IX, XI, XIII	Endocrine / Nutritional / Metabolic system, Circulatory system, Digestive system & Musculoskeletal/connective tissue	556	5.28
IX, X, XI	Circulatory system, Respiratory system, & Digestive system	287	2.73
IV, IX, XIII	Metabolic system, Circulatory system, & Musculoskeletal/connective tissue	269	2.56
X, XI, XIII	Respiratory system, Digestive system & Musculoskeletal/connective tissue	263	2.5
VII, IX, XI	Eye, Circulatory system & Digestive system	234	2.22
IV, XI, XIII	Endocrine / Nutritional / Metabolic system, Digestive system & Musculoskeletal/connective tissue	233	2.22
VIII, IX, XI	Ear/mastoid, Circulatory system, Digestive system	228	2.16
IX, XI, XII	Circulatory system, Digestive system & Skin/subcutaneous tissue	225	2.14
IX, XI, XIV	Circulatory system, Digestive system, & Genitourinary system	162	1.54

IV, VIII, IX, XI	Endocrine / Nutritional / Metabolic system, Ear/mastoid, Circulatory system, Digestive system	142	1.35
IV, VIII, IX, X, XI, XIII	Endocrine / Nutritional / Metabolic system, Ear/mastoid, Circulatory system, Respiratory system, Digestive system & Musculoskeletal/connective tissue	141	1.34
IV, IX, X, XI	Endocrine / Nutritional / Metabolic system, Circulatory system, Respiratory system & Digestive system	139	1.32
VIII, XI, XIII	Ear/mastoid, Digestive system & Musculoskeletal/connective tissue	133	1.27
IV, VII, IX, XI	Endocrine / Nutritional / Metabolic system, Eye, Circulatory system & Digestive system	127	1.2
XI, XII, XIII	Digestive system, Skin/subcutaneous tissue & Musculoskeletal/connective tissue	124	1.18
XI, XIII, XIV	Digestive system, Musculoskeletal/connective tissue & Genitourinary system	112	1.07
VIII, IX, XI, XIII	Ear/mastoid, Circulatory system, Digestive system & Musculoskeletal/connective tissue	112	1.07
IV, IX, XI, XIV	Endocrine / Nutritional / Metabolic system, Circulatory system, Digestive system & Genitourinary system	111	1.06
IX, X, XI, XIII	Circulatory system, Respiratory system, Digestive system & Musculoskeletal/connective tissue	110	1.05

Table S4. Prevalence Risk Ratio (PRR) and Prevalence Risk Differences (PRD) and Adjusted Prevalence Risk Ratio and Adjusted Prevalence Risk Differences of complex multimorbidity across various socio-demographic characteristics.

Correlates		Complex Multimorbidity Present			
		PRR	PRD	APRR	APRD
Age	45-59 years	0.62 (0.56, 0.69)	−0.16 (−0.20, −0.13)	0.69 (0.62, 0.77)	−0.13 (−0.16, −0.09)
	60-74 years	0.94 (0.86, 1.03)	−0.03 (−0.06, 0.01)	0.98 (0.90, 1.08)	−0.01 (−0.05, 0.02)
	75 years and more	Reference			
Gender	Male	0.91 (0.84, 0.99)	−0.03 (−0.06, −0.005)	0.95 (0.88, 1.03)	−0.02 (−0.05, 0.01)
	Female	Reference			
Residence	Rural	Reference			
	Urban	1.13 (1.03, 1.24)	0.04 (0.01, 0.08)	1.10 (1.01, 1.20)	0.04 (0.006, 0.07)
Social Group	Scheduled Tribes	0.92 (0.85, 0.99)	−0.03 (−0.06, −0.004)	1.02 (0.93, 1.11)	−0.001 (−0.03, 0.03)
	Scheduled Castes	0.68 (0.60, 0.77)	−0.12 (−0.15, −0.09)	0.77 (0.67, 0.88)	−0.07 (−0.11, −0.03)
	Other Backward Class	0.93 (0.86, 1.02)	−0.02 (−0.06, 0.01)	0.97 (0.89, 1.07)	−0.01 (−0.04, 0.02)
	Other Castes	Reference			
Education	No Formal Education	1.19 (0.96, 1.49)	0.05 (−0.01, 0.12)	1.16 (0.95, 1.43)	0.06 (−0.004, 0.12)
	Up to Primary	1.32 (1.06, 1.64)	0.09 (0.03, 0.15)	1.30 (1.07, 1.58)	0.10 (0.04, 0.15)

	Middle school to Higher Secondary & Diploma	1.28 (0.996, 1.65)	0.08 (0.003, 0.16)	1.27 (1.02, 1.58)	0.08 (0.01, 0.15)
	Graduation & above	Reference			
**Employment status**	Never Worked	Reference			
	Currently not working	1.08 (0.99, 1.19)	0.03 (−0.004, 0.07)	1.04 (0.96, 1.14)	0.03 (−0.004, 0.06)
	Currently working	0.68 (0.60, 0.76)	−0.13 (−0.16, −0.09)	0.77 (0.69, 0.85)	−0.07 (−0.11, −0.04)
**Wealth Index**	Poorest	0.81 (0.71, 0.92)	−0.07 (−0.12, −0.03)	0.79 (0.69, 0.89)	−0.09 (−0.14, −0.04)
	Poorer	0.84 (0.74, 0.96)	−0.06 (−0.11, 0.01)	0.82 (0.73, 0.92)	−0.07 (−0.12, −0.03)
	Middle	0.85 (0.74, 0.97)	−0.06 (−0.11, −0.009)	0.84 (0.74, 0.95)	−0.07 (−0.11, −0.12)
	Richer	0.97 (0.84, 1.11)	−0.01 (0.07, 0.04)	0.95 (0.83, 1.08)	−0.02, (−0.07, 0.03)
	Richest	Reference			

Table S5. Prevalence Odds Ratio (POR) of complex multimorbidity across various socio-demographic characteristics.

**Correlates**		**Complex Multimorbidity Present**	
		**POR**	**APOR**
**Age**	45-59 years	Reference	
	60-74 years	1.86 (1.62, 2.14)	1.70 (1.48, 1.95)
	75 years and more	2.06 (1.75, 2.43)	1.78 (1.50, 2.11)
**Gender**	Male	Reference	
	Female	1.16 (1.02, 1.31)	1.09 (0.96, 1.23)
**Residence**	Rural	Reference	
	Urban	1.22 (1.05, 1.41)	1.19 (1.03, 1.37)
**Social Group**	Scheduled Tribes	Reference	
	Scheduled Castes	1.53 (1.27, 1.85)	1.51 (1.24, 1.83)
	Other Backward Class	1.57 (1.29, 1.93)	1.42 (1.17, 1.72)
	Other Castes	1.75 (1.47, 2.09)	1.49 (1.22, 1.81)
**Education**	No Formal Education	Reference	
	Up to Primary	1.17 (1.06, 1.29)	1.19 (1.06, 1.34)
	Middle school to Higher Secondary & Diploma	1.12 (0.90, 1.38)	1.12 (0.93, 1.36)
	Graduation & above	0.77 (0.57, 1.05)	0.75 (0.54, 1.04)
**Employment status**	Never Worked	1.78 (1.49, 2.13)	1.45 (1.24, 1.70)
	Currently not working	2.04 (1.79, 2.33)	1.61 (1.42, 1.83)
	Currently working	Reference	
**Wealth Index**	Poorest	Reference	
	Poorer	1.06 (0.92, 1.22)	1.07 (0.93, 1.23)
	Middle	1.08 (0.93, 1.25)	1.10 (0.95, 1.29)
	Richer	1.31 (1.10, 1.56)	1.35 (1.14, 1.60)
	Richest	1.39 (1.12, 1.71)	1.50 (1.21, 1.87)

Table S6. Number of hospital visits among patients with complex multimorbidity and non-complex multimorbidity.

		Mean ± SD
**Number of IDP visits**	Total population (N = 24,073)	0.15 ± 0.57
	Complex Multimorbidity (N = 9113)	0.20 ± 0.67
	Non-complex Multimorbidity (N = 14,960)	0.13 ± 0.49
**Number of ODP visits**	Total population (N = 23,757)	4.31 ±4.90
	Complex Multimorbidity (N = 8998)	5.04 ± 5.46
	Non-complex Multimorbidity (N = 14,759)	3.86 ± 4.47

Table S7. Association between healthcare utilization and complex multimorbidity

**Multimorbidity status**	**Healthcare Utilization**	
	**Inpatient visits * AOR (95% CI)**	**Outpatient visits * AOR (95% CI)**
**Complex Multimorbidity**	1.30 (1.13–1.50)	1.08 (0.90–1.30)
**Non-complex Multimorbidity**	Reference	Reference

* adjusted for age, gender, residence, social group, education, employment status, and wealth index

Table S8. Healthcare expenditure in US$ on multimorbidity vs. complex multimorbidity.

**Type of service availed**	**Multimorbidity Present** Median (IQR)	**Complex Multimorbidity Present** Median (IQR)
**Outpatient visits**	8.64 (3.74–21.60)	11.52 (4.61–26.64)
**Inpatient hospitalization**	144.01 (43.20–432.03)	159.13 (49.97–464.43)

1 US$=69.44 INR on 31st Dec, 2018

Table S9. Healthcare expenditure proportion to Gross Domestic Product (GDP) for patients with multimorbidity vs. complex multimorbidity.

Type of service availed	**Multimorbidity Present** Expenditure percentage to GDP	**Complex Multimorbidity Present** Expenditure percentage to GDP
**Outpatient visits**	0.43 (0.19–1.08)	0.58 (0.23–1.34)
**Inpatient hospitalization**	7.21 (2.16–21.63)	7.97 (2.50–23.26)

Proportion of total GDP, based on GDP per capita, India, 2018 in US$ at 1.35% of GDP for healthcare expenditure = 26.95 US$ per capita.
